# Outcomes of sequential third‐line therapies in patients with refractory overactive bladder

**DOI:** 10.1111/iju.15463

**Published:** 2024-05-02

**Authors:** Po‐Ming Chow, Tyler Trump, Howard B. Goldman

**Affiliations:** ^1^ Glickman Urological Institute Cleveland Clinic Cleveland Ohio USA; ^2^ National Taiwan University Hospital and College of Medicine Taipei Taiwan; ^3^ National Taiwan University Hospital Hsin‐Chu Branch Zhubei City Taiwan

**Keywords:** botulinum toxin, incontinence, overactive bladder, sacral neuromodulation, urgency

## Abstract

**Introduction:**

Sacral neuromodulation (SNM) and onabotulinumtoxinA (BoNTA) injection are third‐line therapies for overactive bladder (OAB). Although the efficacy of each third‐line treatment has been well established in clinical trials, there is far less information about performing one third‐line therapy after the other. Our aim is thus to investigate the outcomes of post‐SNM BoNTA and post‐BoNTA SNM as “second” third‐line treatments.

**Methods:**

We retrospectively reviewed all OAB patients who had both SNM and BoNTA between 2013 and 2022. The primary endpoint was the response rates (>50% improvements) of the second third‐line treatments. Secondary endpoints were the proportion of the patients who achieved total dry, the duration of treatments of patients who had significant responses, and risk factors that are associated with treatment response or duration of treatments.

**Results:**

A total of 172 patients had two third‐line therapies. There were 104 patients who had post‐SNM BoNTA and 68 patients who had post‐BoNTA SNM. In the post‐SNM BoNTA group, 62.5% (65/104) had significant responses after BoNTA treatment. In the post‐BoNTA SNM group, 61.8% (44/68) had significant responses after SNM treatment. The proportions of patients who became dry were 21.2% and 23.5%, respectively. In the post‐SNM BoNTA group, spinal pathology is associated with a lower probability of a significant response (48.9% vs. 73.7%, *p*‐value = 0.0105).

**Conclusions:**

BoNTA or SNM remains a viable option for refractory OAB after patients fail from one another. Spinal pathology is associated with a poorer response of post‐SNM BoNTA.

**Abbreviations & Acronyms**
BoNTA: onabotulinumtoxinA
GRA: global response assessment
MS: multiple sclerosis
PTNS: percutaneous tibial nerve stimulation
SCI: spinal cord injury
SNM: sacral neuromodulation
UTI: urinary tract infection

## INTRODUCTION

Overactive bladder syndrome (OAB) is defined as urinary urgency, usually with urinary frequency and nocturia, and with or without urinary incontinence.^1^ The pathophysiology of OAB is unclear, and the proposed mechanisms include bladder ischemia, chronic inflammation, and urothelial dysfunction. These bladder conditions may result from responses to bladder outlet obstruction, inadequate perfusion, or deregulated innervation.^2^

OAB can be largely classified as neurogenic or idiopathic in etiology. Common causes of neurogenic OAB include spinal cord injury (SCI), multiple sclerosis (MS), and stroke. Those without related neurological diseases are considered idiopathic. Treatments for OAB include: behavioral therapy as first‐line, pharmacologic treatment as second‐line, percutaneous tibial nerve stimulation (PTNS), sacral neuromodulation (SNM), or onabotulinumtoxinA (BoNTA) injection as third‐line, and surgical intervention as fourth‐line.^3^

SNM involves implanting a small device in the upper buttock connected to a lead that is placed near the third sacral nerve. This device sends electrical impulses to the nerve, which modulates bladder function, regulates bladder activity, and improves OAB symptoms. The patient can control the device using a remote control, adjusting the stimulation intensity as needed.^4^ SNM is usually performed in two stages. The first stage is a trial period, during which an electrode is placed and the patient's response to the treatment is evaluated. If the trial is successful, a permanent device is implanted. The most common side effects include mild discomfort or pain at the implantation site, temporary tingling or numbness in the legs or genitals, and occasional temporary changes in bowel function.^5^ In addition to OAB, SNM is also indicated for fecal incontinence and non‐obstructive urinary retention.^6^

Intra‐detrusor BoNTA injection involves injecting botulinum toxin directly into the detrusor muscle of the bladder. This toxin acts as a muscle relaxant and reduces bladder activity by blocking the release of acetylcholine, a neurotransmitter that stimulates muscle contraction. In addition, BoNTA may play a role blocking abnormal afferent sensory information.^7^ Intra‐detrusor BoNTA injection can be performed with or without anesthesia. The procedure typically takes about 10–25 min to complete. The most common side effects include temporary urinary retention, urinary tract infection (UTI), and transient blood in the urine.^8^ The effects of intradetrusor BoNTA injection typically last for several months, after which the procedure is repeated. This treatment is considered an effective option for patients who have not responded to other treatments for OAB.

Surgical intervention includes augmentation cystoplasty, suprapubic cystostomy, and urinary diversion. Urinary diversion is a surgical procedure that bypasses the urine from the lower urinary tract. It is irreversible and therefore is rarely used in OAB patients. As a result, patients with refractory OAB usually receive one or more third‐line therapies as their last treatment option. Although the efficacy of each third‐line treatment has been well established in clinical trials, there is far less information about performing one third‐line therapy after the other. Patients are usually offered another third‐line therapy after the first one fails, but it is unclear whether each treatment works as effectively in the later stage as it does in the earlier stage of OAB management. Our aim is thus to investigate the success rates of post‐SNM BoNTA and post‐BoNTA SNM as “second” third‐line treatments and the risk factors for treatment failures. Success after PTNS failure was not evaluated as there were very few patients who underwent that treatment.

## PATIENTS AND METHODS

This study complies with the Declaration of Helsinki and was performed according to ethics committee approval (IRB: 23‐382). The need to obtain informed patient consent was waived.

We performed a retrospective chart review for OAB patients who had both SNM and BoNTA by three fellowship‐trained urologists between 2013 and 2022. No patients were excluded. We collected clinical data including age, sex, underlying disease, symptom improvement from either failed SNM to BoNTA or failed BoNTA to SNM, and complications. Patients were divided into two groups: one treated with SNM followed by BoNTA (the post‐SNM BoNTA group), and one treated with BoNTA followed by SNM (the post‐BoNTA SNM group).

At our institution, BoNTA is injected using a rigid or flexible cystoscope. We administered 10–30 injections of BoNTA (Botox, Allergan, USA), 10 U/1 mL injections about 1 cm apart and 2 mm deep in the detrusor, sparing the trigone.^3^ SNM is performed according to the ICS best practice statement. Bilateral nerve roots were usually evaluated at the trial phase in order to obtain the best response. Unilateral lead is placed for permanent leads.

The primary endpoint was the response rates of the second third‐line treatments. Symptoms before and after second third‐line treatments were compared. The improvement is defined by modified global response assessment (GRA), namely no improvement (0%), mild improvement (1%–50%), moderate improvement (51%–75%), and marked improvement (>75%). The extent of improvement is based on composite symptoms, which are evaluated by both bladder diary and verbal feedback from the patients. Those who had >50% improvements were defined to have significant responses. The original GRA scale has been shown to correlate well with bladder diary and urodynamic studies.^9^ Secondary endpoints were the proportion of the patients who achieved total dry, the duration of treatment of patients who had significant responses, and risk factors that are associated with treatment response or duration of treatments. Stroke indicates cerebral ischemia only. Spinal pathology included spondylolisthesis, spinal canal stenosis, traumatic injury, and disc prolapse. Urinary retention in the setting of post‐treatment complication is defined as one or more episodes that require catheterization, excluding those who were already catheterizing before third‐line therapies. UTI was defined as a positive urine culture with urinary symptoms.

The generalized linear regression model was used to evaluate the risk factors for significant responses. Kaplan–Meier curve with log‐rank test was used to estimate the duration of treatments. Cox proportional hazard model was used for risk factor analysis for the discontinuation of treatments. Statistical tests were performed with R 4.0.0. All statistics were two‐tailed, with *p* < 0.05 as significant.

## RESULTS

In the study period, 1022 patients underwent initial third‐line treatment with SNM and 1117 with BoNTA (see Table S1 for breakdown between neurogenic and non‐neurogenic in each). Among 1022 patients who received SNM first, 104 (10.1%) shifted to BoNTA. Among 1117 patients who received BoNTA first, 68 (6.1%) shifted to SNM. The mean ages for the two groups were 62.6 and 65.9 years old. There were 93 (89.4%) and 62 (91.2%) women in each group. The baseline characteristics was similar between the two groups, with 70 (67.3%) and 49 (72.1%) of them having neurological diseases (Table 1). Among those with spinal pathology, the CIC rates at baseline were 10.6% (5/47) and 17.5% (7/40) for post‐SNM BoNTA and post‐BoNTA SNM, respectively. The median (IQR) follow‐up time of post‐SNM BoNTA (*n* = 104) patients was 12.1 (6.9–26.7) months. The mean (SD) number of injections was 3.4 (3.0) times. For those who received 2 or more injections (*n* = 69), the median (IQR) interval of injection was 5.1 (3.7–8.2) months. The median (IQR) follow‐up time of post‐ BoNTA SNM (*n* = 68) patients was 21.3 (7.1–38.6) months.

**TABLE 1:** Baseline characteristics.

	Post‐SNM BoNTA (*n* = 104)		Post‐BoNTA SNM (*n* = 68)		*p*‐Value
Age (mean, SD)	64.1	14.4	65.93936	13.4945	0.4020
Sex (female)	93	89.4%	62	91.2%	0.9081
Diabetes	42	40.4%	18	26.5%	0.0876
Hypertension	80	76.9%	47	69.1%	0.3364
Coronary heart disease	29	27.9%	18	26.5%	0.9773
Chronic kidney disease	28	26.9%	15	22.1%	0.5890
Stroke	10	9.6%	3	4.4%	0.3334
Dementia	5	4.8%	5	7.4%	0.5190
Parkinson's disease	4	3.8%	4	5.9%	0.7140
Traumatic brain injury	3	2.9%	5	7.4%	0.2670
Spinal pathology	47	45.2%	40	58.8%	0.1113
Multiple sclerosis	5	4.8%	11	16.2%	**0.0250**
Neurogenic bladder	70	67.3%	49	72.1%	0.6235
Fecal incontinence	22	21.2%	16	23.5%	0.8578
SUI	29	27.9%	0	0.0%	**<0.0001**
Prior urethral sling	19	18.3%	0	0.0%	**<0.0001**
Clean intermittent catheterization	12	11.5%	9	13.2%	0.7396
Concomitant overactive bladder medication	53	51.0%	27	39.7%	0.1478
Level of spinal pathology					
Cervical	15	31.9%	15	37.5%	0.6720
Lumbar	31	66.0%	23	57.5%	
Sacral	1	2.1%	2	5.0%	

Abbreviations: BoNTA, onabotulinumtoxinA; SNM, sacral neuromodulation; SUI, stress urinary incontinence.

*Note*: Bold values indicate significance of *p* < 0.05.

In the post‐SNM BoNTA group, 62.5% (65/104) of patients had significant responses after BoNTA treatment. In the post‐BoNTA SNM group, 61.8% (44/68) of patients had significant responses after SNM treatment. The proportions of patients who became dry were 21.2% and 23.5%, respectively. The success rates of neurogenic and non‐neurogenic patients are similar across groups. (Table 2). In the post‐SNM BoNTA group, spinal pathology is associated with less probability of a significant response (48.9% vs. 73.7%, *p* = 0.0105). There were no factors that predicted significant responses in the post‐BoNTA SNM group. The response and duration of prior treatments were not associated with the outcomes of later treatments. In the current cohort, there were 74, 24, and 6 patients who received 100, 200, and 300 U of BoNTA, respectively. The dose of post‐SNM BoNTA did not affect the treatment response in the logistic regression model (Table 3).

**TABLE 2:** Outcome of treatments.

All					
Improvement	Post‐SNM BoNTA (*n* = 104)		Post‐BoNTA SNM (*n* = 68)		*p*‐Value
0	26	25.0%	19	27.9%	0.8850
1%–50%	13	12.5%	7	10.3%	
51%–75%	38	36.5%	22	32.4%	
>75%	27	26.0%	20	29.4%	
Total dry					
No	82	78.8%	52	76.5%	0.7120
Yes	22	21.2%	16	23.5%	

Neurogenic					
	Post‐SNM BoNTA (*n* = 70)		Post‐BoNTA SNM (*n* = 49)		*p*‐Value
Improvement					
0	18	25.7%	12	24.5%	0.7760
1%–50%	11	15.7%	6	12.2%	
51%–75%	24	34.3%	15	30.6%	
>75%	17	24.3%	16	32.7%	
Total dry					
No	57	81.4%	34	69.4%	0.1921
Yes	13	18.6%	15	30.6%	

Non‐neurogenic					
Improvement	Post‐SNM BoNTA (*n* = 34)		Post‐BoNTA SNM (*n* = 19)		*p*‐Value
0	8	23.5%	7	36.8%	0.8040
1%–50%	2	5.9%	1	5.3%	
51%–75%	14	41.2%	7	36.8%	
>75%	10	29.4%	4	21.1%	
Total dry					
No	25	73.5%	18	94.7%	0.0756
Yes	9	26.5%	1	5.3%	

Abbreviations: BoNTA, onabotulinumtoxinA; SNM, sacral neuromodulation.

**TABLE 3:** Factors for >50% improvement from each treatment.

	Post‐SNM BoNTA (*n* = 104)				Post‐BoNTA SNM (*n* = 68)			
	OR	Lower 95%CI	Upper 95%CI	*p*‐Value	OR	Lower 95%CI	Upper 95%CI	*p*‐Value
Age	0.98	0.95	1.01	0.1110	0.98	0.94	1.02	0.3020
Sex (male)	0.30	0.08	1.10	0.0695	0.28	0.05	1.62	0.1540
BMI	1.02	0.97	1.07	0.4470	1.04	0.98	1.11	0.1740
Diabetes	0.81	0.36	1.81	0.6060	2.75	0.79	9.54	0.1110
Hypertension	0.62	0.23	1.66	0.3390	1.32	0.46	3.78	0.6010
Coronary heart disease	0.98	0.40	2.36	0.9550	0.96	0.32	2.91	0.9470
Chronic kidney disease	0.60	0.25	1.45	0.2560	0.91	0.28	2.94	0.8730
Stroke	0.89	0.24	3.37	0.8640	1.25	0.11	14.50	0.8580
Dementia	0.38	0.06	2.39	0.3030	0.38	0.06	2.47	0.3130
Parkinson's disease	0.19	0.02	1.87	0.1540	0.60	0.08	4.54	0.6210
Spinal pathology	**0.34**	0.15	0.78	**0.0105**	1.08	0.40	2.91	0.8810
Multiple sclerosis	0.90	0.14	5.61	0.9060	1.80	0.43	7.53	0.4180
Neurogenic bladder	0.59	0.25	1.42	0.2370	1.25	0.43	3.69	0.6830
Fecal incontinence	1.37	0.50	3.73	0.5360	0.74	0.24	2.31	0.6040
BoNTA dose	1	0.992	1.01	0.8290				

Abbreviations: BoNTA, onabotulinumtoxinA; SNM, sacral neuromodulation.

*Note*: Bold values indicate significance of *p* < 0.05 and the corresponding odds ratio (OR).

**FIGURE 1:**
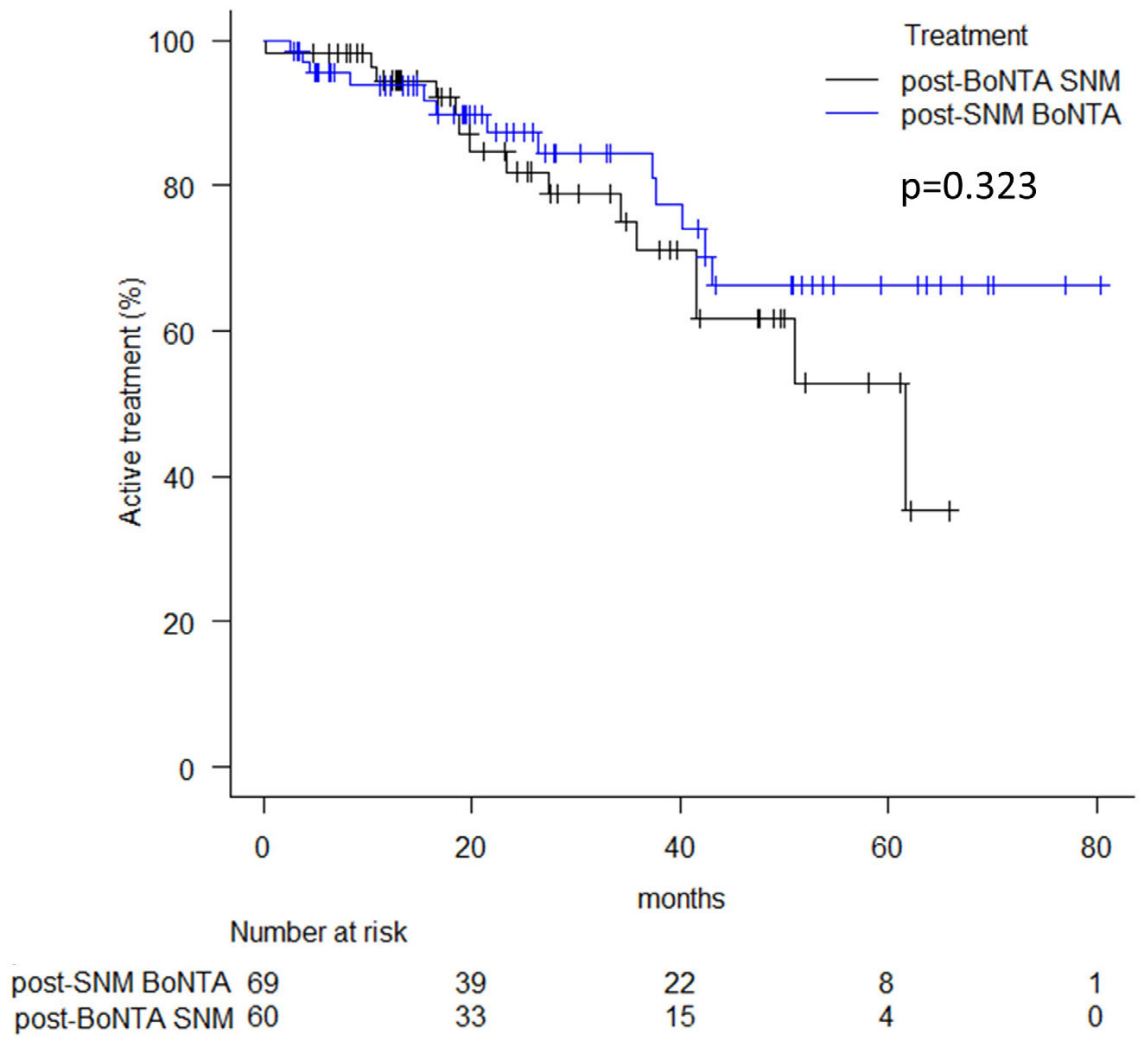
Treatment duration (months) of patients who had >1 onabotulinumtoxinA (BoNTA) injection and who had stage 2 sacral neuromodulation (SNM).

For 69 patients who had more than one injection in the post‐SNM BoNTA group, 71.8% continued on BoNTA injection. The median duration of treatment has not been met (Figure 1). There were no factors identified to be associated with the duration of treatment (Table S2). For 60 patients who had permanent implant of SNM, 15 of them failed (13 patients left the inactive device in situ, and two patients had the device removed) at the end of follow‐up (Figure 1). Men were associated with a shorter duration of treatment (Table S3).

There were 19 (18.3%) cases of urinary retention and 22 (21.2%) UTIs from post‐SNM BoNTA. All were managed with catheterization or antibiotics. There was one constipation and one severe pain from post‐BoNTA SNM, resulting in device removal. There was no difference in retention between those with neurologic disease (17.9%) and those without (19.2%).

## DISCUSSION

Our study found that post‐SNM BoNTA and post‐BoNTA SNM both have greater than 60% success rates when used after the other has failed in patients with refractory OAB syndrome. For patients who have undergone >1 BoNTA injection or have reached stage 2 SNM, more than 50% of them retain their therapy at 5 years. The responses to prior therapy did not influence the improvement from or the duration of the later therapy. Spinal pathology was associated with less improvement in post‐SNM BoNTA patients. This is the first study reporting the outcomes of two third‐line therapies in different sequences.

The overall success rate of SNM is constantly >50% in large clinical trials. The success rate is 50%–76%^10^, ^11^, ^12^ in neurogenic patients and 56%–90% in non‐neurogenic patients.^5^, ^13^, ^14^ An early meta‐analysis of SNM for neurogenic patients reported pooled success rates of 68% for the test phase and 92% for permanent SNM.^15^ A more recent meta‐analysis reported similar pooled success rates of 66.2% for the test phase and 84.2% for permanent SNM in neurogenic patients.^16^ The success rate of SNM after prior BoNTA has been reported in small retrospective series, and a pooled analysis reported a 58% success rate in 319 patients with refractory OAB. Although the success rates varied with different definitions in these studies, they are generally lower than SNM without prior BoNTA.^17^

Among the 60 post‐BoNTA SNM patients who had permanent implants of SNM, there were only five men. The duration of active SNM of the five men ranged from 10.3 to 28.3 months. Although the duration of treatment was significantly shorter, the number of male patients was too small to make robust inferences. Some prior studies reported higher success rates in women, while others reported gender not being predictive. A review indicated differences in neurochemical regulation in brain, anatomical structure, and sex hormones to account for the sexual differences in bladder dysfunction.^18^ One Medicare study proposed that the long‐term effects of prostatic obstruction contribute to overall worse outcome of SNM in men.^19^ Whether there is a true gender discrepancy in post‐BoNTA SNM or there is potential interaction between gender and other factors requires further investigation.

The overall success rate of BoNTA was >60% in phase three trials.^20^, ^21^, ^22^, ^23^ The success rate was 87.6%–92.1% in neurogenic patients^24^ and 74%–83% in non‐neurogenic patients.^25^ Treatment duration depends on efficacy and the tolerability to AE including AUR and UTI, and is potentially limited by the development of neutralizing antibodies to BoNTA after repeat injection.^26^ Because of its less invasive nature, BoNTA is often offered by some prior to SNM after failed medical therapy. The success rate of post‐SNM BoNTA is therefore rarely reported. There has been only one study in idiopathic OAB patients, which reported a 43% success rate.^27^ The definition of success was based on the bladder diary of the prior study but was based on subjective improvement in our study, and the difference in definition could be the source of different results.

Our study is the first to report the impact of spinal pathology on post‐SNM BoNTA. In prior reports of BoNTA in SCI patients, the success rates were 64.2%,^28^ compared to 50%–91% in patients with stroke^29^, ^30^ and 79.2%–100% in patients with Parkinson's disease.^31^, ^32^ A large phase three trial reported similar results in patients with SCI and MS,^21^ with the success rate around 75%. A small series reported 91% success in SCI and 50% in stroke patients.^30^ However, none of these studies focused on post‐SNM patients. Although the underlying mechanism is still unclear, our finding suggests that lack of response to SNM in SCI patients could be predictive of less efficacy of BoNTA injection (48.9% and 73.7% response rate for patients with and without spinal pathology, respectively). One possible reason could be the bladder condition is poorer in this late stage of disease.

There are several limitations to our study. First, our study lacks the severity of symptoms at baseline, making it difficult to compare the outcomes between the two groups. Second, the outcome was measured only by subjective improvement, and no urodynamic or bladder diary data were included for analysis. Third, the dosage of BoNTA was not the same for the entire cohort. The dosage ranged from 100 U to 300 U per injection in our cohort, but was not associated with response rates in our study. Despite these limitations, our study provides unprecedented data regarding long‐term outcomes of sequential third‐line therapies for OAB. BoNTA or SNM remains a viable option for refractory OAB after patients fail the other.

In conclusion, more than 60% of patients who have either post‐BoNTA SNM or post‐SNM BoNTA have >50% improvement in their overall symptoms. For patients who have undergone >1 BoNTA injection or have reached stage 2 SNM, more than 50% of them stay on the same therapy at 5‐year follow‐up. Spinal pathology is associated with a poorer response of post‐SNM BoNTA.

## AUTHOR CONTRIBUTIONS

**Po‐Ming Chow:** Conceptualization; methodology; software; data curation; investigation; validation; formal analysis; visualization; writing—original draft. **Tyler Trump:** Data curation; investigation. **Howard B. Goldman:** Conceptualization; investigation; validation; supervision; writing—review & editing; resources; project administration.

## CONFLICT OF INTEREST STATEMENT

Dr. Howard Goldman declares the following conflict of interests: Medtronic—consultant and grant support. Bluewind—consultant. BrightUro—consultant. Boston Scientific—consultant. Cook—study participant. Laborie—consultant. Neuspera—consultant. NewUro—consultant. Urovant—consultant.

## APPROVAL OF THE RESEARCH PROTOCOL BY AN INSTITUTIONAL REVIEWER BOARD

Cleveland Clinic IRB: 23‐382.

## INFORMED CONSENT

Not applicable.

## REGISTRY AND THE REGISTRATION NO. OF THE STUDY/TRIAL

Not applicable.

## ANIMAL STUDIES

Not applicable.

## Supporting information


Table S1.

Table S2.

Table S3.
